# Environmental Conditions as Determinants of Kidney Stone Formation

**DOI:** 10.1021/acsabm.3c00722

**Published:** 2023-11-01

**Authors:** Carmen González-Enguita, Gonzalo Bueno-Serrano, Andrés López de Alda-González, Rosario García-Giménez

**Affiliations:** † Hospital Universitario Fundación Jiménez Díaz, Avenida Reyes Católicos, 2, 28040 Madrid, Spain; ‡ Hospital Don Benito, Avenida Primero de Mayo, 25, 06400 Don Benito, Badajoz, Spain; § Departamento de Geología y Geoquímica, Facultad de Ciencias, Universidad Autónoma, 28049 Madrid, Spain

**Keywords:** kidney stone, oxalate, struvite, rural/urban area, Don Benito (Badajoz), Madrid, Spain

## Abstract

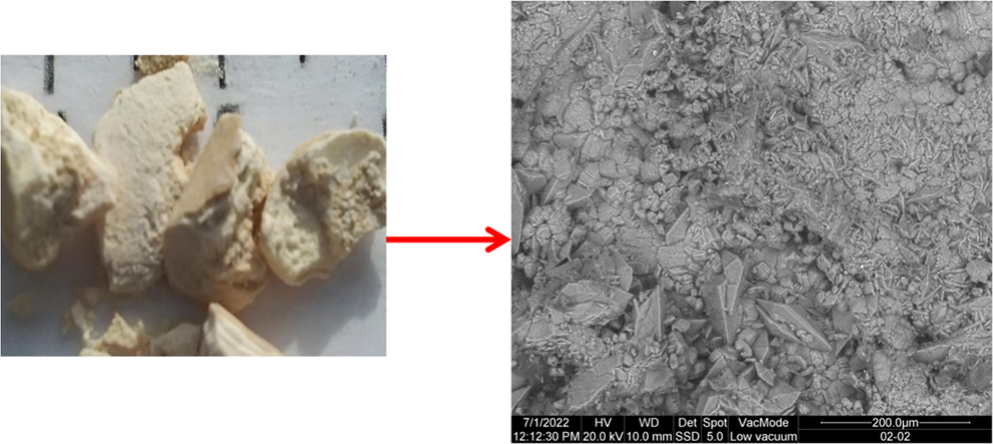

Urolithiasis is a disease characterized by the presence of stones in the urinary tract, whether in the kidneys, ureters, or bladder. Its origin is multiple, and causes can be cited as hereditary, environmental, dietary, anatomical, metabolic, or infectious factors. A kidney stone is a biomaterial that originates inside the urinary tract, following the principles of crystalline growth, and in most cases, it cannot be eliminated naturally. In this work, 40 calculi from the Don Benito, Badajoz University Hospital are studied and compared with those collected in Madrid to establish differences between both populations with the same pathology and located in very different geographical areas. Analysis by cathodoluminescence offers information on the low crystallinity of the phases and their hydration states, as well as the importance of the bonds with the Ca cation in all of the structures, which, in turn, is related to environmental and social factors of different population groups such as a high intake of proteins, medications, bacterial factors, or possible contamination with greenhouse gases, among other factors.

## 1 Introduction

Urolithiasis is a disease characterized by the presence of stones in the urinary tract, whether in the kidneys, ureters, or bladder. Its origin is multiple, and causes can be cited as hereditary, environmental, dietary, anatomical, metabolic, or infectious factors.^1−3^ There are records that are more than 1000 years old related to urinary lithiasis. Hippocrates possessed an understanding about the disease and its characteristic symptoms in depth; there are even records of surgical procedures performed in antiquity.^4^ The first study on the composition of a kidney stone was carried out around the year 1800 by Schellee and Bergman, chemicals and pharmaceuticals, who identified a uric acid stone.

A kidney stone (urolithiasis) is a biomaterial originating in the urinary tract following the principles of crystalline growth and, in most cases, cannot be eliminated naturally. This fact occurs mainly because the appropriate environment for the nucleation and subsequent formation of a germ is provided by crystal deposits around a crystalline nucleus and due to the difficulty in detecting their presence until there are clinical symptoms or they are large enough to be detected by imaging techniques.^5^

For the characterization of kidney stones, different analytical techniques have been employed, the pioneer among them being IR spectroscopy^6^ and also Fourier transform infrared spectroscopy (FT-IR)^7,8^ that classify the results based on groups of compounds (uricite, oxalates, phosphates), and small crystalline or amorphous organic compounds have also been identified.^9^ Of great importance are X-ray images;^10−13^ techniques such as scanning electron microscopy (SEM) together with energy-dispersive X-ray spectroscopy (EDX) and thermogravimetry (TGA) help us understand the nature of kidney stones,^14,15^ in addition to computed tomography (TC).^15−17^

Physical techniques help in elemental analysis such as total reflection X-ray fluorescence (TXRF or TRXRF),^18^ laser-induced decay spectroscopy (LIBS),^19,20^ and inductively coupled plasma mass spectrometry by laser ablation (LA-ICP-MS) just to name a few. Thus, various chemical elements have been identified, with some belonging to phosphate class minerals and others falling into the organic class, such as oxalates, very frequently encountered in kidney stones.^12^ Raman spectroscopy has also been applied, which allows the identification of organic and inorganic compounds,^21−23^ cathodoluminescence and thermoluminescence.^24^ Biochemical methods have been the primary analytical techniques used to characterize the chemical composition of kidney stones.^25,26^

The main phases found in kidney stones are monohydrated and dihydrated oxalates, whewellite (CaC_2_O_4_·H_2_O) and weddellite (CaC_2_O_4_·2H_2_O), respectively; phosphates such as brushite (Ca(PO_3_OH)·2H_2_O), whitlockite (Ca_9_Mg(PO_3_OH)(PO_4_)_6_), struvite (Mg(NH_4_)(PO_4_)·6H_2_O), hydroxyapatite (Ca_5_(PO_4_)_3_(OH)), and hydroxyapatite carbonates (Ca_5_(PO_4_)_*x*_(CO_3_)_3–*x*_(OH)); and other compounds derived from uric acid, such as uricite, which is anhydrous uric acid (C_5_H_4_N_4_O_3_), or dihydrate (C_5_H_4_N_4_O_3_·2H_2_O) or ammonium urate (NH_4_C_5_H_3_N_4_O_3_).^27^ Given such a variety of phases, mineral components are generally divided into three groups: oxalates, phosphates, and purines.

Worldwide, analyses related to the composition of kidney stones have been carried out in major regions: Europe,^28,29^ Asia,^30,31^ USA,^32,33^ Mexico,^34^ Africa,^35−37^ and India.^38^ The percentage incidence of renal lithiasis differs greatly in different parts of the world: in Asia it is 1–5%, in Europe it is 5–9%, in North America it is 13–15%, and in Saudi Arabia it is approximately 18–20%. In Spain, the prevalence rate is higher than 4% and, specifically, it rises to 14.3% in the Balearic Islands.

In Morocco, for example, according to some research, there are no up-to-date studies reporting the full prevalence of calcium oxalate throughout the country. However, there are only some statistics on calcium oxalate in some regions, especially in Rabat-Sale and Fez-Meknes with values of 66.6 and 60.98%, respectively.^39^

Kidney stones in which cystine appears (SCH_2_CH(NH_2_)CO_2_H)_2_ are rare. Cystine stones are produced by an inherited disorder of the transport of amino acid cystine that results in more than cystine in the urine.^40^

In this paper, 40 kidney stones are studied—22 of them were from the Don Benito, Badajoz University Hospital and 18 were from the Fundación Jiménez Daz (Madrid)—to establish differences between both populations with the same pathology and located in two very different regions in Spain.

## 2 Materials and Methods

### 2.1 Materials

We analyzed 22 samples of kidney stones from the Don Benito Hospital of Badajoz with different shades, all of them with sequential crystalline growths whose original point is a crystallization nucleus and overlapping but perfectly identifiable layers. In general, the external zone of the kidney stones presents a reddish color and is less compact than the internal one. Don Benito is a municipality in the province of Badajoz, Spain, with a population of 37,310 inhabitants and a density of 66.42 inhabitants/km^2^; most of them reside in the urban center and work in the service sector, which, together with the food industry, comprise the main source of the city’s vibrancy. The kidney stones were procured from the San Antonio hospital, a reference center for many inhabitants of the entire region belonging to different social statuses. However, the population can be considered to be of rural origin.

Madrid is the capital of Spain. It has a little more than three million registered inhabitants. The samples collected from the Fundación Jiménez Diaz University Hospital predominantly represent middle-class urban residents, totaling 18 samples with varying morphologies, compactness, and colors, although they mostly have reddish tones, with some exhibiting zoning. All kidney stones, extracted in both Don Benito (Badajoz) and Madrid, were obtained from surgical interventions in which the stones were removed.

### 2.2 Methods

Sample mineralogy was analyzed with powder X-ray microdiffraction (XRD) on a PAN Analytical X’Pert Pro X-ray diffractometer fitted with a Cu anode. The operating conditions were 40 mA, 45 kV, divergence slit of 0.5°, and 0.5 mm reception slits. The powder samples were scanned with a step size of 0.0167 (2θ) at 150 ms per step and 2θ angles of 5–60°. The detected phases were identified using the Crystallography Open Database (COD) library of crystal structures.

The microscopy and chemical analyses as well as the cathodoluminescence (CL) measurements were performed by scanning electron microscopy and energy-dispersive X-ray spectroscopy (SEM-EDS) using an Inspect-S ESEM instrument from the FEI Company.

Raman spectra of the samples were carried out by means of a Thermo-Fisher DXR Raman microscope (West Palm Beach, FL 33407) with a point-and-shoot Raman capability of 1 μm spatial resolution using a laser source at 532 nm.

CL spectra were prepared on polished slabs under a low vacuum mode without coating to maintain an open pathway for the CL emission, using a Gatan MonoCL3 detector with a PA-3 photomultiplier attached to the ESEM. The PMT covers a spectral range of 185–850 nm and is the most sensitive in the blue parts of the spectrum. A retractable parabolic diamond mirror and a photomultiplier tube are used to collect and amplify the luminescence signal. The sample was positioned ∼10 mm beneath the bottom of the CL mirror assembly. The excitation for CL measurements was provided at a 20 kV electron beam.

## 3 Results and Discussion

### 3.1 By Powder X-ray Microdiffraction (XRD)

**Figure 1:**
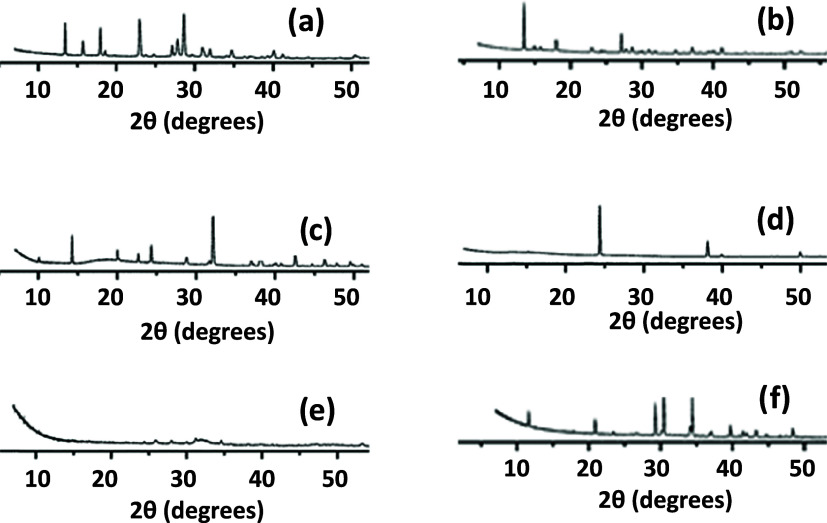
X-ray microdiffraction analyses: Badajoz Hospital: (a) uricite, (c) whewellite, and (e) calcium magnesium phosphate. Madrid Hospital: (b) uricite, (d) weddellite, (f) struvite.

The analysis of X-ray microdiffraction on samples allowed the identification of major phases in the studied kidney stones, which are presented in Figure 1. The mineral phases that have been mostly identified in the samples from Madrid and Badajoz are listed in Table 1.

**Table 1:** Abundance of Phases in the Studied Kidney Stones from Badajoz and Madrid Cities

phases	Badajoz Hospital kidney stone (%)	Madrid Hospital kidney stone (%)
uricine	10	30
whewellite	50	40
wheddellite	15	5
cystine	0	5
phosphates	25	20

The most abundant phases in both populations are listed in Table 1. The predominant composition in both cities was whewellite, accounting for nearly 50%, with the notable absence of cystine and a scarcity of uric acid stones in the kidney stones from Badajoz. Phosphates were abundant in both locations, representing a quarter of the sample. In Madrid, calcium-based phosphates (apatite Ca_5_(PO_4_)_3_(OH) and brushite CaHPO_4_·2H_2_O), calcium–magnesium-based phosphates (whitlockite Ca_9_Mg(PO_4_)_6_(PO_3_OH)), and ammonium-based (struvite NH_4_MgPO_4_. 6H_2_O) stones were identified. Hydroxyfluorapatite, hydroxyapatite, and calcium–magnesium hydrogen apatite were found in kidney stones from Badajoz. The nonexistence of struvite in the studied Badajoz stones is striking. The presence of phosphates is conditioned by pH.^41^ Struvite is a common compound that contributes significantly to water contamination in large cities.^42,43^

Uric acid and the corresponding urates act as essential biomineral phases in kidney stones, serving as crystalline seeds upon which different phases develop in successive crystallization stages.

Uric acid and its corresponding salts have been widely described^39^ and constitute, in most kidney stones, the germ of the biomineral nucleation process. Uric acid is a biomineral that constitutes the main crystallization nuclei, generating epitaxial growths. Anhydrous uric acid or uricite^8^ is the most thermodynamically stable form and is also the most frequent.

Oxalates, both monohydrate (whewellite) and dihydrate (weddellite), are very common biominerals that precipitate when urine is supersaturated with calcium, depositing on already crystallized materials such as urates and forming crystals with specific morphologies whose crystallization directs the urates into a heterogeneous nucleation process.^37^ The two oxalates described, given the simple difference of presenting a varied number of water molecules, can undergo conversion by a reversible drying/hydration process, with the monohydrate being the most stable phase.^44^ Lastly, cystine is a relatively uncommon phase that forms at acidic pH levels.^45^

### 3.2 By Scanning Electron Microscopy and Energy-Dispersive X-ray Spectroscopy (SEM-EDS) Analyses

**Figure 2:**
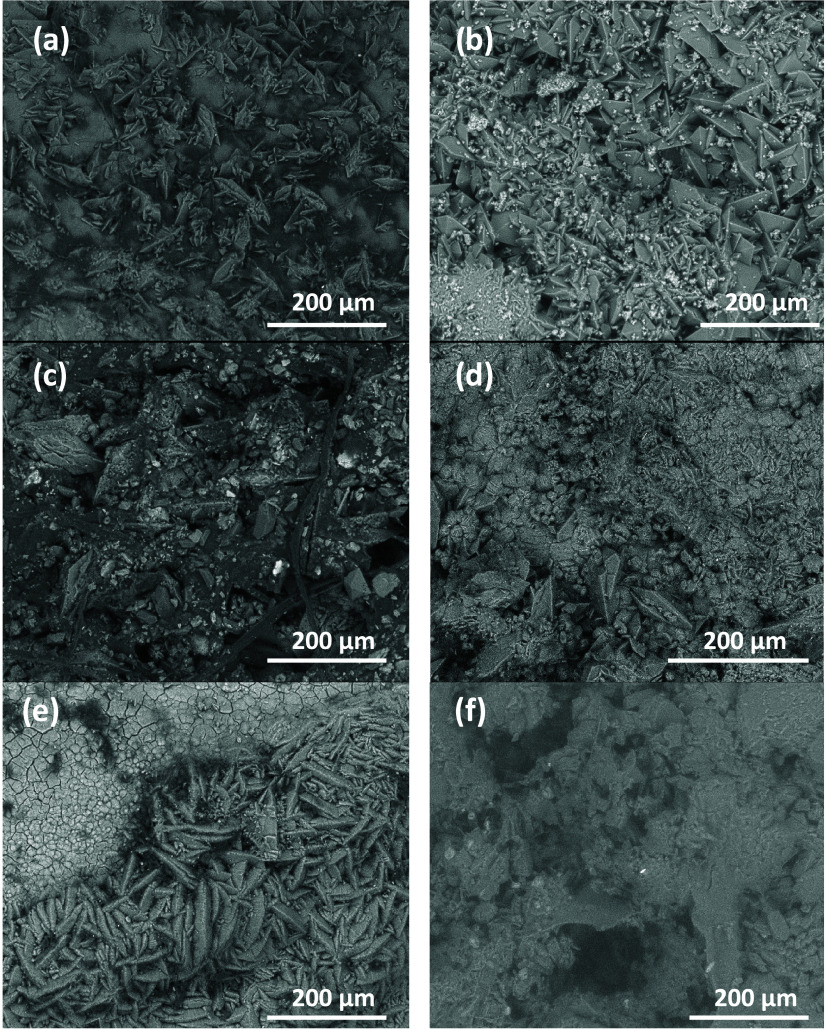
SEM-EDS analyses: Badajoz Hospital: (a) whewellite, (c) weddellite and uricite, (e) left, phosphate; right, whewellite. Madrid Hospital: (b) whewellite, (d) uricite, and (f) phosphate.

Figure 2 shows some of the images of the different kidney stones studied. Figure 2a,b shows images of calcium oxalate monohydrate (whewellite), which is usually present in most stones with its typical monoclinic prisms and clear exfoliation, indicating a higher pseudorhombic symmetry. The crystals are in a banded arrangement, suggesting a homogeneous nucleation due to the high supersaturation in the medium and which might be suspended when other substances with sizes less than the critical size appear in the system or due to competition in the available space to crystallize.

Figure 2c represents a kidney stone from Badajoz, and it presents a chaotic morphology corresponding to several crystalline phases that represent a heterogeneous nucleation based on oxalate. This type of nucleation is simpler than homogeneous nucleation since it only requires the presence of solid particles that can attract and retain on their surface the species that will constitute the eventual crystal. On the oxalate, in this case hydrated (weddellite, characterized by its tetragonal pyramids), uricite crystallizes together with organic matter. Once the nucleus is formed, subsequent crystal formation involves the combination of two processes: crystal growth and aggregation. Figure 2d shows the monoclinic prisms of the uricite, in this case, from a kidney stone in a Madrid Hospital; however, those identified from the Badajoz Hospital were also identical. Figure 2e displays the phosphatic formations (hexagonal morphology) present in the stones of the two cities, and in both cases, they participate in a heterogeneous nucleation with growth zonation. Phosphates are a group of biominerals typical of living organisms that can occur in various phases and with different cations, the most common being calcium, although magnesium or ammonium can also appear in their compositions. Figure 2e shows the phosphate phase with a hexagonal morphology in short prisms.

Figure 2f shows a struvite phosphate with a rhombic morphology. This phase is one of the most harmful in wastewater, and in large cities, it creates problems associated with its treatment and introduction into the circular economy.^46−48^ The results obtained from the samples are in agreement with those obtained by X-ray microdiffraction analyses.

Cystine, an organosulfur amino acid compound with a hexagonal morphology and overlapping growths in layers and homogeneous nucleation, has been identified in a small sample of stones from Madrid. The crystals aggregate in a specific pattern, forming the characteristic morphology of cystine stones, which is rare in kidney stones.^49^ The chemical composition of kidney stones has been found to be closely related to various factors related to lifestyle, diet, and medication.

### 3.3 By Raman Spectroscopy Analyses

**Figure 3:**
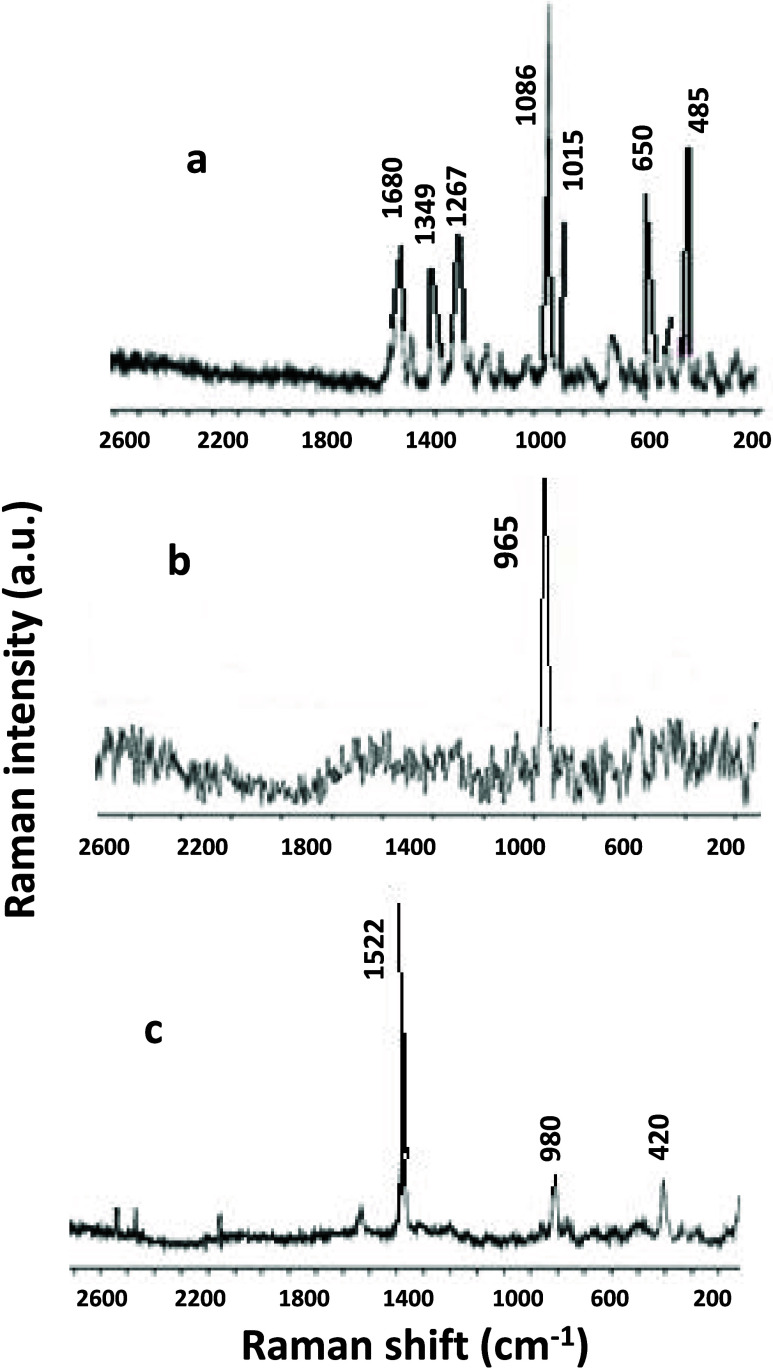
Raman spectroscopy analyses: (a) uricite, (b) tricalcium phosphate, and (c) calcium oxalate hydrate.

Raman spectroscopy was used to identify the mineral components of the kidney stones in this work (Figure 3). Three Raman spectra corresponding to samples rich in uricite, tricalcium phosphate, and calcium oxalate hydrate have been presented, which have been analyzed by comparison with similar standards.

Figure 3a presents typical Raman bands of uric acid, with three of moderate intensity: at 1685/1700 cm^–1^ corresponding to the C=O stretching band of the carbonyl group, at 1580/1620 cm^–1^ relative to the stretching band in the carbon–carbon bonds, and at 1330/1360 cm^–1^ relative to the stretching of the carbon–nitrogen bond (C–N), and finally a band of weak intensity at 860/900 cm^–1^ corresponding to the bending of the carbon–hydrogen bonds.^50^Figure 3b shows the Raman spectrum with typical bands of tricalcium phosphate with medium intensity at 420/450 cm^–1^, corresponding to Ca–O stretching; at 560/600 cm^–1^ for the angular deformation of the phosphate group; and another at 1030/1060 cm^–1^ for the stretching of the P–O bond. In addition, the P–O stretching band is identified, which is the most intense in the spectrum at 940/970 cm^–1^^51^.^51^

Finally, Figure 3c presents the Raman spectrum of calcium oxalate featuring the band at (a) 1480/1580 cm^–1^ relative to the stretching of the C–C bonds of the oxalate anion having a high intensity; (b) 1320/1390 cm^–1^ relative to the stretching vibration band of the carbonyl group (C=O) in the oxalate anion having a low intensity; (c) 750/800 cm^–1^ relative to the C–O flexion band having a moderate intensity; and (d) 470/560 cm^–1^ relative to the stretching of the calcium–oxygen (Ca–O) bonds in the crystalline calcium oxalate lattice having a medium intensity.^52^

### 3.4 By Cathodoluminescence (CL) Analyses

**Figure 4:**
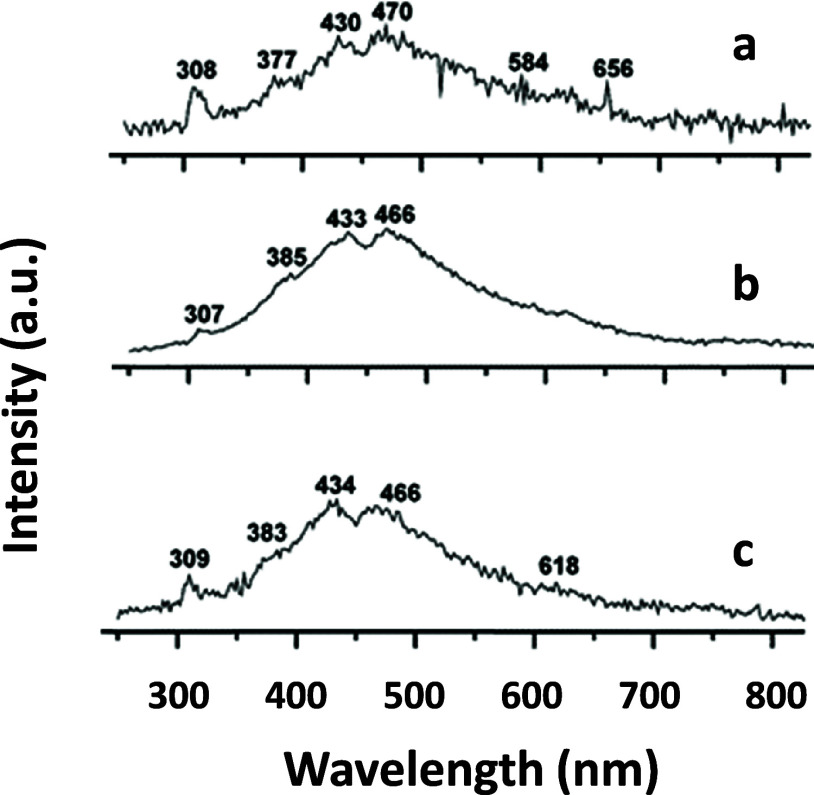
CL analyses: (a) weddellite, (b) CaMgHPO_4_, and (c) whewellite.

Figure 4 shows CL analyses of some of the phases present in the studied kidney stones corresponding to stones rich in weddellite, whitlockite, and whewellite. Each of the samples studied differs significantly in terms of both chemical and structural properties.

Weddellite and whewellite (Figure 4a,c) are two calcium oxalate minerals that frequently appear in kidney stones. Their crystal structures are similar; both have a monoclinic symmetry and a crystal lattice with alternating layers of calcium ions and oxalate molecules. However, there are differences in the arrangement of the oxalate ions in the two phases. Weddellite has a more ordered structure with oxalate ions arranged head-to-tail, while whewellite has a more disordered structure with oxalate ions arranged head-to-head and tail-to-tail. All this translates into different physical and chemical properties, such as solubility and reactivity and, of course, in the formation of kidney stones.^53,54^

Magnesium whitlockite (Figure 4b) and β-tricalcium phosphate are terms that are used interchangeably as it is difficult to distinguish the two phases by XRD analyses.^55^ Magnesium whitlockite is found primarily in association with the pathologic mineralization of various soft tissues and stones. The characterization techniques capable of unequivocally distinguishing between different calcium phosphate phases are high-resolution imaging, crystallography, and/or spectroscopy as well as CL, which is a poorly crystalline apatite in which calcium ions are replaced by others existing in the human tract. Mg whitlockite is a pathological biomineral.^55^

All of the CL spectra collected, although different, have low-intensity emission spectra with poorly defined bands related to the low crystallinity of the phases. The bands in the blue UV region from 200 to 480 nm are related to vacancies (intrinsic defects), linear or structural defects, and defects related to dehydroxylation, dehydration, or very frequent chemical reactions within the human body; in the 500/850 nm range, green infrared, it is related to extrinsic defects.

## 4 Conclusions

The description of the composition of kidney stones by means of different analytical techniques including XRD, SEM-EDS, Raman spectroscopy, and CL, collected from surgical interventions in two cities with very different lifestyles in Spain, Don Benito (Badajoz) and Madrid, can provide valuable information about the causes of the formation of kidney stones and try to generate protocols for their prevention.

The composition of the stones can lead to different treatments of the wastewaters of the two mentioned cities, which, of course, have different phases, especially in the wastewater treatment plants in relation to the presence of struvite in them. The variety of phases in kidney stones allows the identification of compounds, such as uricite, cystine, whitlockite, weddellite, whewellite, and struvite. SEM-EDS offers insights into the surface morphology and chemical composition, which are somewhat dispersed with infrequent but not significant chemical elements for their classification.

The techniques used in the analysis are complementary, and the less frequent Raman and CL techniques provide more precise information on the chemical and structural composition of kidney stones. These techniques can provide additional information about the crystallographic and molecular structures of the minerals present in the stones, identifying trace elements and impurities, and determining the chemical bonds and molecular structures of kidney stones. They are complementary techniques that explain more precise structural models.

CL provides information on the poor crystallinity of the phases and their hydration states (hydroxyl groups) as well as on the importance of bonds with the Ca cation in all structures. All of this is related to environmental and social factors of different population groups such as a high protein intake (see uricite), medications or bacterial factors (see struvite), and possible contamination with greenhouse gases (see cystine).

In general, the kidney stones procured from Badajoz Hospital are zonal stones, with the internal zone being clearer than the external one. In Madrid Hospital, there was a greater variety: they were zoned and had a single color. Regarding composition, the zonation in the stones responds to the same components; that is, the different states of crystallization are similar to varied aggregation situations that induce the crystallization of the same species.

The composition of kidney stones is very similar in both locations. The biggest difference is the presence of struvite in the calculi from Madrid. The nonexistence of struvite in kidney stones procured from the Badajoz Hospital means that this compound is barely detected in the sewage network of this city and lacks the environmental problems related to its elimination.
